# Supplementary material for: Ciprofloxacin-Loaded Mixed Polymeric Micelles as Antibiofilm Agents
Source: Pharmaceutics. 2023 Apr 4;15(4):1147. doi: 10.3390/pharmaceutics15041147 (PMC10145464; doi:10.3390/pharmaceutics15041147)
Supplement: Supplementary file 1 [file pharmaceutics-15-01147-s001.zip › pharmaceutics-2306217-supplementary.pdf]

Supplementary Materials: Ciprofloxacin-Loaded Mixed Polymeric Micelles as Antibiofilm Agents

Rumena Stancheva, Tsvetelina Paunova-Krasteva, Tanya Topouzova-Hristova, Stoyanka Stoitsova and Petar Petrov Emi Haladjova,

Supplementary information:

1. Preparation of mixed micelles

Calculation of hydrophilic-lipophilic balance (HLB) of copolymers

For HLB calculation the Griffin method [1] was used following the equation:

$HLB = 20 * M_h/M$

where M_h is the molar mass of the hydrophilic block of copolymer, and M is the molecular mass of the whole macromolecule.

Determination of critical micellization concentration (CMC)

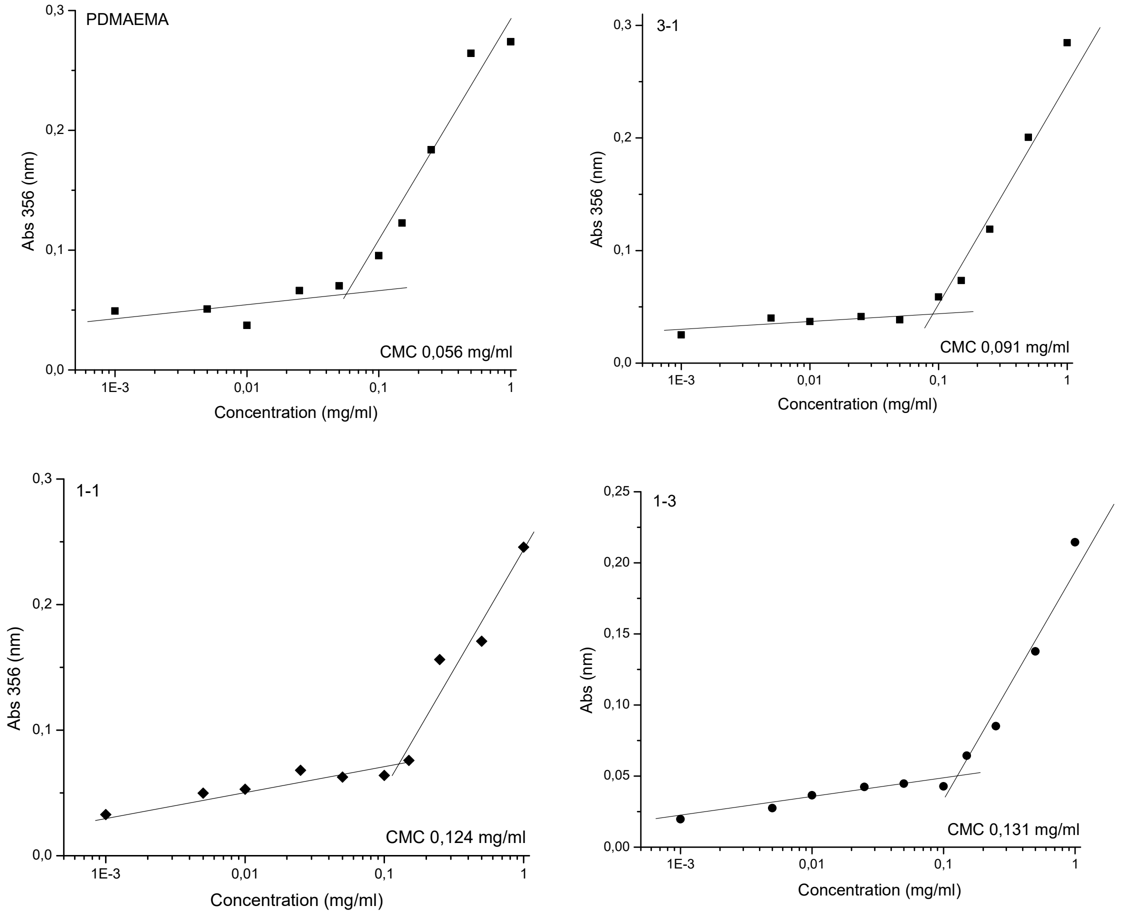

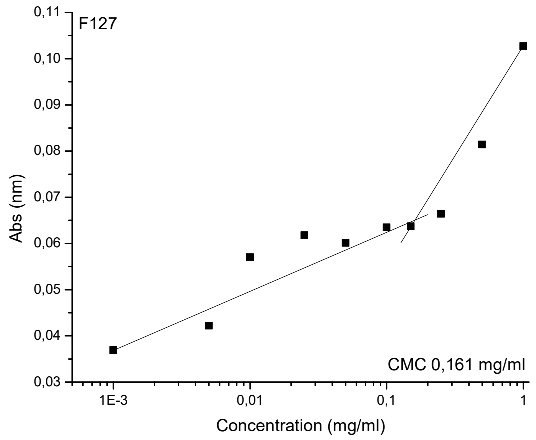

Figure S1. Critical micelle concentration plot of SCPMs and MPMs based on PDMAEMA-b-PCL-b-PDMAEMA and Pluronic F127 triblock copolymers.

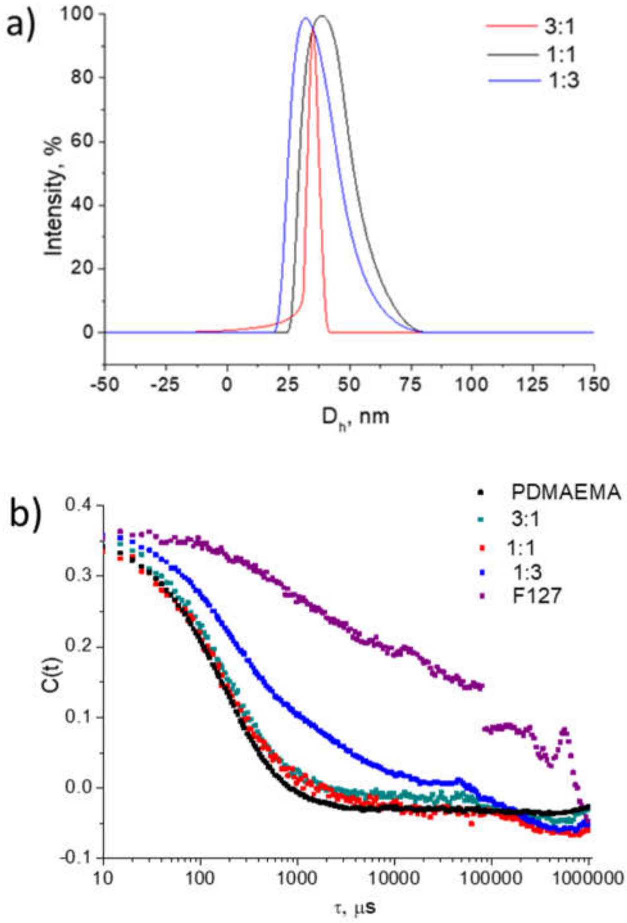

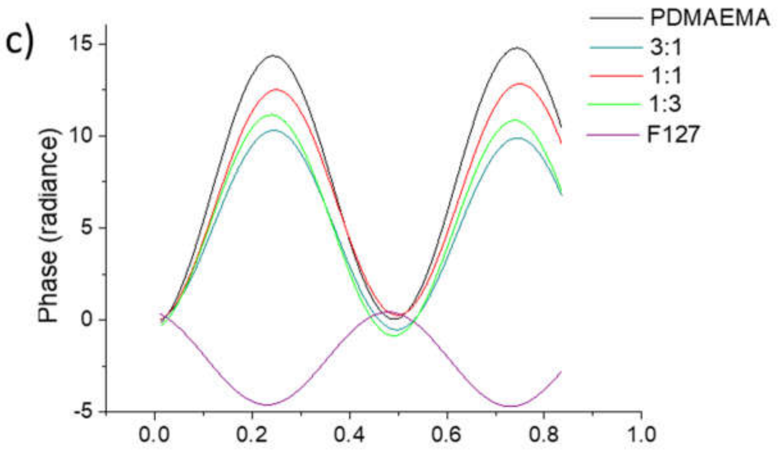

Figure S2. Size distribution curves (a), correlation curves (b) and phase plots (c) of SCPMs and MPMs based on PDMAEMA-b-PCL-b-PDMAEMA and Pluronic F127 triblock copolymers at concentration 0.01 mg.ml^{-1}.

2. Loading of micelles with CF

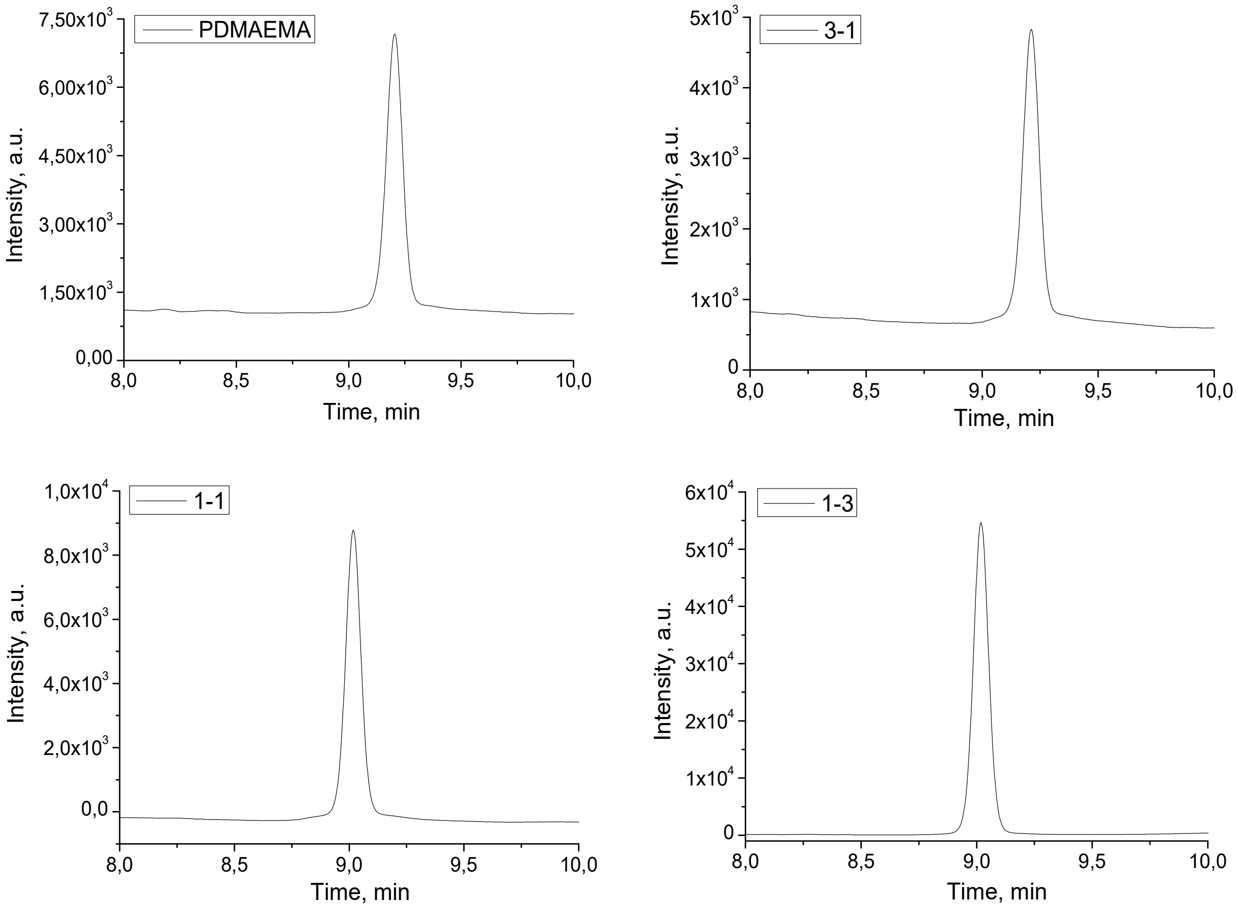

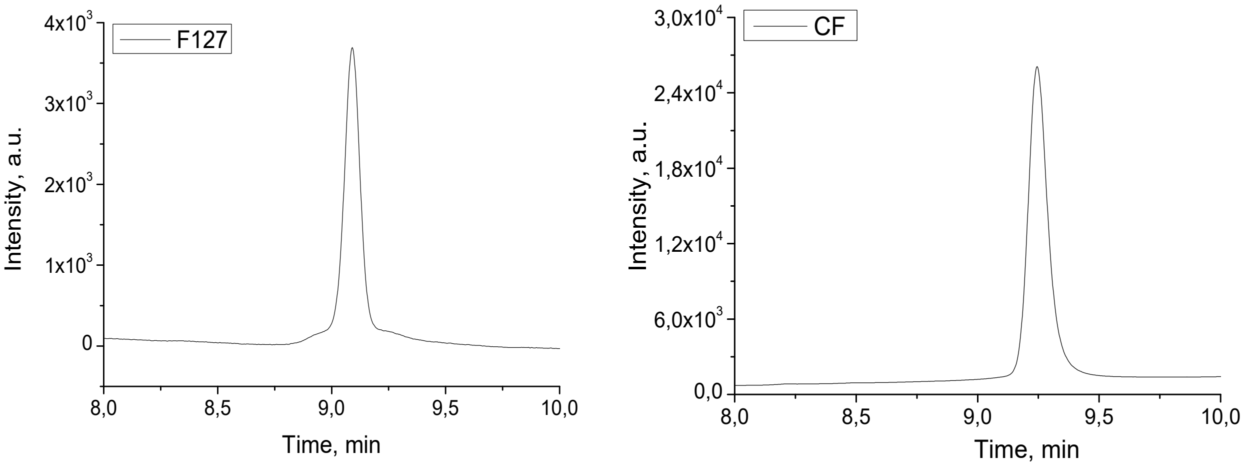

Figure S3. HPLC chromatograms of non-loaded (free) CF extracted from SCPMs and MPMs based on PDMAEMA-b-PCL-b-PDMAEMA and Pluronic F127 triblock copolymers. HPLC chromatogram of pure CF used as standard is given as well. The samples were prepared at polymer to drug mass ratio 10:1. The integrated area of the peaks was used for calculation of EE and DLC.

Table S1. Encapsulation efficiency (%) towards CF, determined by HPLC and UV spectrophotometry, of SCPMs and MPMs based on PDMAEMA-b-PCL-b-PDMAEMA and Pluronic F127 triblock copolymers prepared at different polymer to drug mass ratio. Each value represents the arithmetic mean of three separate experiments.

**Polymeric micelles**	**PDMAEMA**		**3:1**		**1:1**		**1:3**		**F127**	
**Polymer to drug ratio**	**HPLC**	**UV**	**HPLC**	**UV**	**HPLC**	**UV**	**HPLC**	**UV**	**HPLC**	**UV**
50:1	85.6 ±4.3	90.9 ±4.6	98.9 ±2.9	97.7 ±3.3	87.3 ±5.2	90.2 ±4.5	97.9 ±2.4	97.0 ±2.8	99.0 ±1.8	96.4 ±3.8
10:1	98.1 ±4.1	98.7 ±3.6	98.8 ±2.5	99.0 ±2.1	99.2 ±1.6	99.2 ±2.2	99.4 ±2.1	99.1 ±1.9	98.6 ±1.8	91.2 ±2.2
5:1	74.8 ±4.8	80.1 ±3.6	86.9 ±4.3	92.5 ±4.1	84.9 ±3.8	90.7 ±4.1	92.1 ±4.5	94.2 ±4.9	82.2 ±4.3	73.0 ±4.2
2.5:1	48.9 ±5.0	59.13 ±5.1	51.2 ±5.2	64.1 ±5.9	52.9 ±5.2	69.6 ±6.1	62.0 ±6.0	70.3 ±5.8	31,0 ±4.6	30.2 ±4.8
1:1	42.8 ±4.3	68.0 ±5.9	54.4 ±5.2	74.6 ±6.9	63.0 ±6.0	70.6 ±6.2	57.7 ±6.1	78.6 ±6.9	43.4 ±4.3	41.0 ±4.0

Table S2. Drug loading content (%) towards CF, determined by HPLC and UV spectrophotometry, of SCPMs and MPMs based on PDMAEMA-b-PCL-b-PDMAEMA and Pluronic F127 triblock copolymers prepared at different polymer to drug mass ratio. Each value represents the arithmetic mean of three separate experiments.

**Polymeric micelles**	**PDMAEMA**		**3:1**		**1:1**		**1:3**		**F127**	
**Polymer to drug ratio**	HPLC	UV	HPLC	UV	HPLC	UV	HPLC	UV	HPLC	UV
50:1	2.1 ±0.2	2.2 ±0.1	2.0 ±0.2	2.0 ±0.3	1.8 ±0.1	1.9 ±0.1	2.2 ±0.1	2.1 ±0.2	2.0 ±0.1	1.9 ±0.2
10:1	10.4 ±0.8	10.5 ±0.4	11.0 ±0.5	10.9 ±0.5	10.9 ±0.4	10.9 ±0.3	10.3 ±0.4	10.3 ±0.9	9.9 ±0.8	9.3 ±0.7
5:1	15.6 ±1.1	17.0 ±0.9	17.9 ±0.6	18.7 ±0.9	17.5 ±0.8	18.7 ±0.6	19.2 ±0.8	19.6 ±0.9	16.4 ±0.9	14.6 ±0.7
2.5:1	20.6 ±1.9	25.0 ±1.0	21.2 ±1.0	26.6 ±0.9	21.9 ±0.9	28.8 ±1.1	26.3 ±0.7	29.8 ±1.2	12.4 ±1.3	12.4 ±1.2
1:1	44.5 ±1.3	70.1 ±1.2	56.6 ±1.3	74.4 ±1.5	65.5 ±1.2	73.8 ±1.2	59.4 ±1.4	80.3 ±1.5	43.4 ±1.8	41.0 ±1.6

3. CF release from micelles

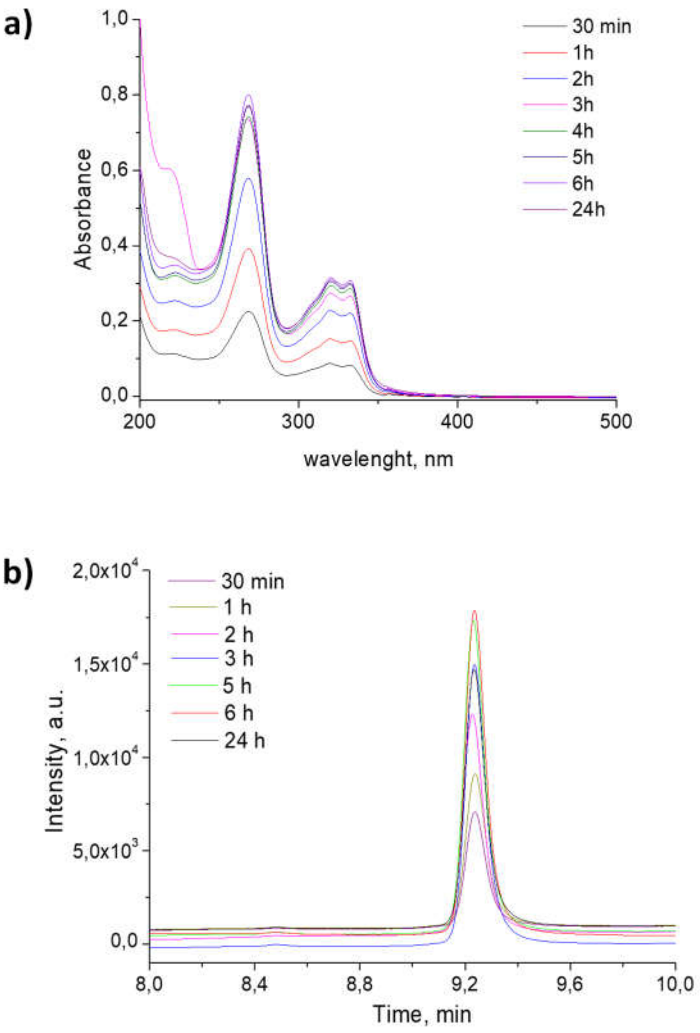

Figure S4. UV-Vis absorption spectra (a) and HPLC chromatograms (b) of CF released from SCPMs and MPMs based on PDMAEMA-b-PCL-b-PDMAEMA and Pluronic F127 triblock copolymers at 37 °C in phosphate buffer pH 7.4.

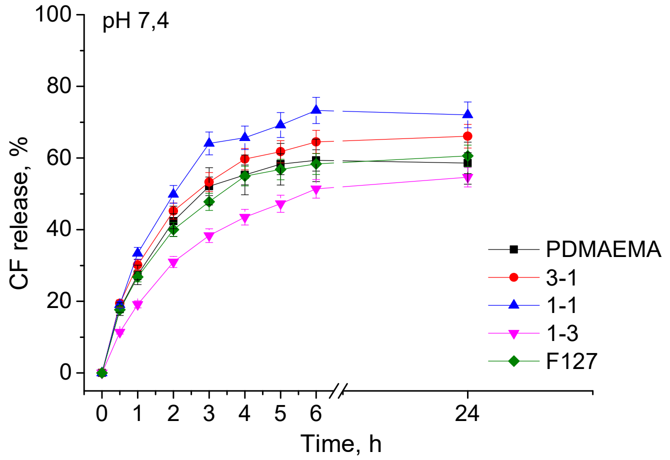

Figure S5. CF release profiles from SCPMs and MPMs based on PDMAEMA-b-PCL-b-PDMAEMA and Pluronic F127 triblock copolymers prepared at 10:1 polymer to drug mass ratio determined from UV-Vis spectrophotometry. The release was performed at 37 °C in phosphate buffer pH 7.4. Each data point in represents the arithmetic mean ± SD of three separate experiments.

References:

1. Griffin WC: "Calculation of HLB Values of Non-Ionic Surfactants," Journal of the Society of Cosmetic Chemists 5 (1954): 259.
